# Functionally enhanced placenta-derived mesenchymal stem cells inhibit adipogenesis in orbital fibroblasts with Graves’ ophthalmopathy

**DOI:** 10.1186/s13287-020-01982-3

**Published:** 2020-11-05

**Authors:** Jae Yeon Kim, Sohae Park, Hyun-Jung Lee, Helen Lew, Gi Jin Kim

**Affiliations:** 1 Department of Biomedical Science, CHA University, Seongnam, 13488 Republic of Korea; 2 Center for Non-Clinical Development, CHA Advanced Research Institute CHA University, Seongnam, 13488 Republic of Korea; 3 Department of Ophthalmology, CHA Bundang Medical Center CHA University, Seongnam, 13496 Republic of Korea

**Keywords:** Adipogenesis, Graves’ ophthalmopathy, Gene modification, Phosphatase of regenerating liver-1, Placenta-derived mesenchymal stem cells

## Abstract

**Background:**

Placenta-derived mesenchymal stem cells (PD-MSCs) have unique immunomodulatory properties. Phosphatase of regenerating liver-1 (PRL-1) regulates the self-renewal ability of stem cells and promotes proliferation. Graves’ ophthalmopathy (GO) is an autoimmune inflammatory disease of the orbit and is characterized by increased orbital levels of adipose tissue. Here, we evaluated the therapeutic mechanism for regulation of adipogenesis by PRL-1-overexpressing PD-MSCs (PD-MSCs^PRL-1^, PRL-1+) in orbital fibroblast (OF) with GO patients.

**Methods:**

PD-MSCs isolated from human placenta were transfected with the PRL-1 gene using nonviral transfection method. Primary OFs were isolated from orbital adipose tissue specimens from GO patients. After maturation as adipogenic differentiation, normal and GO-derived OFs were cocultured with naïve and PD-MSCs^PRL-1^. We analyzed the protein levels of adipogenesis markers and their signaling pathways in OFs from GO patients.

**Results:**

The characteristics of PD-MSCs^PRL-1^ were similar to those of naïve cells. OFs from GO patients induced adipocyte differentiation and had significantly decreased a lipid accumulation after coculture with PD-MSCs^PRL-1^ compared to naïve cells. The mRNA and protein expression of adipogenic markers was decreased in PD-MSCs^PRL-1^. Insulin-like growth factor-binding proteins (IGFBPs) secreting PD-MSCs^PRL-1^ downregulated the phosphorylated PI3K/AKT/mTOR expression in OFs from GO patients. Interestingly, IGFBP2, − 4, − 6, and − 7 expression in PD-MSCs^PRL-1^, which was mediated by integrin alpha 4 (ITGA4) and beta 7 (ITGB7), was higher than that in naïve cells and upregulated phosphorylated FAK downstream factor.

**Conclusion:**

In summary, IGFBPs secreting PD-MSC^PRL-1^ inhibit adipogenesis in OFs from GO patients by upregulating phosphorylated FAK and downregulating PI3K/AKT/mTOR signaling pathway. The functional enhancement of PD-MSCs by nonviral gene modification provides a novel therapeutic strategy for the treatment of degenerative diseases.

## Background

Graves’ ophthalmopathy (GO) is a thyroid-associated autoimmune disease of the eye that is potentially sight-threatening. The main symptoms of GO are proptosis-associated impairment of eye motility, lid retraction, de novo adipogenesis, and soft tissue inflammation. In particular, inflammatory reactions of orbital fibroblasts (OFs) are responsible for these disease symptoms [1]. Substantial evidence suggests the involvement of insulin-like growth factor 1 receptor (IGF-1R) in GO [2]. Thyroid-stimulating hormone receptor (TSHR) and IGF-1R, which are OF surface receptors, stimulate hyaluronic acid synthesis and de novo adipogenesis through peroxisome proliferator-activated receptor gamma (PPAR-γ) [3, 4].

Based on the molecular pathogenesis of GO, medical and surgical treatments of patients with GO have been implemented. In particular, corticosteroids and orbital radiotherapy continue to be used to treat patients with GO [5]. Orbital radiotherapy combined with corticosteroids protects against disease progression by reducing compressive optic neuropathy in patients with active thyroid eye disease [6]. However, glucocorticoid therapy has a negative effect on patient hyperthyroid status and adrenal insufficiency, as well as acute liver damage, when alanine aminotransferase levels are greater than 300 U/L [7, 8]. Moreover, medical radiotherapy also resulted in the development of malignancies, depending on the age and gender of patients [9].

Placenta-derived mesenchymal stem cells (PD-MSCs) have been broadly investigated due to their multilineage differentiation potential, and these cells have especially potent immunomodulatory abilities associated with tissue repair and regenerative medicine. MSCs inhibit the proliferation of T, B, natural killer, and dendritic cells. Due to these immunoregulatory properties, the safety and clinical efficacy of MSC-based therapy has tested in preclinical [10] and transplantation studies [11]. In comparison to other MSCs, PD-MSCs have an additional immunomodulatory advantage by regulating the expression of human leukocyte antigen (HLA)-ABC and HLA-G [12]. Therefore, the therapeutic effects of PD-MSCs are considered to be associated with immunosuppression-mediated replacement of damaged tissues.

Phosphatase of regenerating liver-1 (PRL-1), also known as protein tyrosine phosphatase type IVA member 1 (PTP4A1) and PTPCAAX1, is a member of a small class of prenylated PTPs. PRL-1 was originally identified as an immediate early gene during liver regeneration [13]. PRL-1 contains the C-terminal prenylation motif for farnesylation CAAX [14]. PRL-1 promotes cellular proliferation during protein prenylation, which is a posttranslational lipid modification, by upregulating RhoA via the mevalonate metabolic pathway. The major enzyme β-hydroxy β-methylglutaryl-coenzyme A (HMG-CoA) reductase regulates AMP-activated protein kinase (AMPK) during protein prenylation through PRL-1 [15]. Moreover, PRL-1 modulates the oxidative stress response in the mammalian retina [16]. PRL-2, which is in the same class and subfamily as PRL-1, plays an important role in hematopoietic stem cell self-renewal.

Recently, we reported therapeutic effects of naïve PD-MSCs in mice models of GO [17]. However, it is still unknown whether functionally enhanced PD-MSCs overexpressing PRL-1 (PD-MSCs^PRL-1^, PRL-1+) inhibit adipogenesis in OFs from GO patients to investigate therapeutic effects.

## Materials and methods

### Cell culture and gene transfection

Orbital adipose tissue specimens were obtained from patients with GO (*n* = 3) during fat decompression and from control individuals without a history of GO (*n* = 3) under consent conditions. OF preparation was approved by the Institutional Review Board of CHA Bundang Medical Center, Seongnam, Republic of Korea (IRB-2018-01-007). Orbital tissue explants were minced and treated with 0.25 mg/mL collagenase (Sigma-Aldrich, St. Louis, MO, USA) for 1 h at 37 °C in a shaking incubator. After collagenase digestion, the orbital tissues were placed in culture plates with DMEM/F12 supplemented with 20% fetal bovine serum (FBS; Gibco, Carlsbad, CA, USA) and 1% penicillin/streptomycin (P/S; Gibco).

Placentas were collected for research purposes by the Institutional Review Board of CHA Gangnam Medical Center, Seoul, Republic of Korea (IRB 07–18). All participants provided written informed consent prior to placenta collection. PD-MSCs were isolated as previously described [18] and cultured in α-modified minimal essential medium (α-MEM; HyClone Logan, UT, USA) supplemented with 10% FBS (Gibco), 1% P/S (Gibco), 1 μg/mL heparin (Sigma-Aldrich), and 25 ng/mL human fibroblast growth factor-4 (hFGF-4; Peprotech, Rocky Hill, NJ, USA). The PRL-1 plasmid vector was purchased from Origene (#RG200435; Rockville, MD, USA). To induce overexpression of the PRL-1 gene, naïve PD-MSCs (passage = 7) were transfected using the AMAXA nucleofector system (Lonza, Basel, Switzerland) according to the manufacturer’s instructions. After transfection for 24 h, the cells were selected by 1.5 mg/mL neomycin. All cells were maintained at 37 °C in a humidified atmosphere containing 5% CO_2_.

### Differentiation of PD-MSCs^PRL-1^ and OFs from GO patients

To analyze the potential of PD-MSCs^PRL-1^ to differentiate into mesodermal lineages, PD-MSCs^PRL-1^ (passage = 5) were plated at a density of 5 × 10^3^ cells/cm^2^ in various differentiation induction media using the StemPro adipogenesis and osteogenesis differentiation kit (Gibco) according to the manufacturer’s instructions. After approximately 21 days, PD-MSCs^PRL-1^ were fixed in 4% paraformaldehyde and incubated for 1 h with Oil Red O (Sigma-Aldrich) to stain lipids to visualize lipid vesicles and von Kossa with 5% silver nitrate (Sigma-Aldrich) under the light to evaluate the accumulation of calcium deposits.

To induce adipogenic differentiation, normal and GO-derived OFs (5 × 10^3^ cells/cm^2^) were seeded and incubated in serum-free DMEM/F12 supplemented with 33 μM biotin, 17 μM pantothenic acid, 10 μg/mL transferrin, 0.2 nM triiodothyronine (T_3_), 1 μM insulin (all from Sigma-Aldrich), 0.2 μM carbaprostacyclin (cPGI_2_; Cayman Chemical, Ann Arbor, MI, USA), 1 μM dexamethasone, and 0.1 mM isobutylmethylxanthine (IBMX; all from Sigma-Aldrich) for the first 4 days. To induce the maturation of adipocytes, the medium was supplemented except 1 μM dexamethasone and 0.1 mM IBMX (all from Sigma-Aldrich) for 6 days and was replaced every other day. Lipid accumulation and adipocyte morphology were visualized by Oil Red O staining.

### Coculture experiments

To detect the inhibition of adipogenesis, normal and GO-derived OFs underwent adipogenic differentiation and were cocultured with naïve and PD-MSCs^PRL-1^ (5 × 10^3^ cells/cm^2^) in Transwell inserts (8 μm pore size; Corning, NY, USA) in α-MEM (HyClone) supplemented with 10% FBS and 1% P/S (all from Gibco) for 24 h at 37 °C in a humidified atmosphere containing 5% CO_2_.

### Reverse transcription polymerase chain reaction (RT-PCR) and quantitative real-time PCR (qRT-PCR)

Total RNA was extracted using TRIzol LS reagent (Invitrogen, Carlsbad, CA, USA) according to the manufacturer’s protocol. The concentration and purity of the total RNA were determined spectrophotometrically by measuring the ODs at 260 nm and 280 nm. cDNA was reverse transcribed from total RNA (500 ng) by using SuperScript III reverse transcriptase (Invitrogen). To analyze stemness markers in PD-MSCs^PRL-1^, PCR amplification was performed with specific primers (Table 1). β-actin was used as an internal control. The amplified PCR products were electrophoresed on 2% agarose gels containing 1.5 μg/mL ethidium bromide and visualized under UV light. qRT-PCR analysis was used to determine differences in gene expression. qRT-PCR was performed with primers (Table 2) and SYBR Green PCR master mix (Roche, Basel, Switzerland) in a CFX Connect™ Real-Time System (Bio-Rad, Hercules, CA, USA). All reactions were performed in triplicate.

**Table 1:** Primer sequences using reverse transcription polymerase chain reaction

Genes		Primer sequences	Tm
Oct4	Forward	5′-AGTGAGAGGCAACCTGGAGA-3’	52
	Reverse	5′-GTGAAGTGAGGGCTCCCATA-3’	
Nanog	Forward	5′-TTCTTGACTGGGACCTTGTC-3’	52
	Reverse	5′-GCTTGCCTTGCTTTGAAGCA-3’	
Sox2	Forward	5′-GGGCAGCGTGTACTTATCCT-3’	52
	Reverse	5′-AGAACCCCAAGATGCACAAC-3’	
HLA-G	Forward	5′-GCGGCTACTACAACCAGAGC-3’	58
	Reverse	5′-GCACATGGCACGTGTATCTC-3’	
TERT	Forward	5′-GAGCTGACGTGGAAGATGAG-3’	55
	Reverse	5′-CTTCAAGTGCTGTCTGATTCCAATG-3’	
AFP	Forward	5′-ATGCTGCAAACTGACCACGC-3’	55
	Reverse	5′-GCTTCGCTTTGCCAATGCTT-3’	
Albumin	Forward	5′-TGAGTTTGCAGAAGTTTCCA-3’	60
	Reverse	5′-CCTTTGCCTCAGCATAGTTT-3’	
β-actin	Forward	5′-TCCTTCTGCATCCTGTCAGCA-3’	58
	Reverse	5′-CAGGAGATGGCCACTGCCGCA-3’

**Table 2:** Primer sequences using quantitative real time polymerase chain reaction

Genes		Primer sequences	Tm
OC	Forward	5′-AGTGAGAGGCAACCTGGAGA-3’	52
	Reverse	5′-GTGAAGTGAGGGCTCCCATA-3’	
COL1A1	Forward	5′-TTCTTGACTGGGACCTTGTC-3’	52
	Reverse	5′-GCTTGCCTTGCTTTGAAGCA-3’	
Adipsin	Forward	5′-GGGCAGCGTGTACTTATCCT-3’	52
	Reverse	5′-AGAACCCCAAGATGCACAAC-3’	
PPAR-γ	Forward	5′-GCGGCTACTACAACCAGAGC-3’	58
	Reverse	5′-GCACATGGCACGTGTATCTC-3’	
Adiponectin	Forward	5′-GAGCTGACGTGGAAGATGAG-3’	55
	Reverse	5′-CTTCAAGTGCTGTCTGATTCCAATG-3’	
Leptin	Forward	5′-ATGCTGCAAACTGACCACGC-3’	55
	Reverse	5′-GCTTCGCTTTGCCAATGCTT-3’	
LPL	Forward	5′-TGAGTTTGCAGAAGTTTCCA-3’	60
	Reverse	5′-CCTTTGCCTCAGCATAGTTT-3’	
FABP4	Forward	5′-GCATGGCCAAACCTAACATGA-3’	55
	Reverse	5′-CCTGGCCCAGTATGAAGGAAA-3’	
IGFBP1	Forward	5′-GAGCCCTGCCGAATAGAAC-3’	60
	Reverse	5′-GGATCCTCTTCCCATTCCAAG-3’	
IGFBP2	Forward	5′-ACATCCCCAACTGTGACAAG-3’	60
	Reverse	5′-ATCAGCTTCCCGGTGTTG-3’	
IGFBP3	Forward	5′-CAGAGCACAGATACCCAGAAC-3’	60
	Reverse	5′-AGCACATTGAGGAACTTCAGG-3’	
IGFBP4	Forward	5′-CTGACAGCTTTCGAGAGTGAG-3’	60
	Reverse	5′-GCGCATTTGAGGGAAACTTC-3’	
IGFBP5	Forward	5′-ACCCAGTCCAAGTTTGTCG-3’	60
	Reverse	5′-TGTAGAATCCTTTGCGGTCAC-3’	
IGFBP6	Forward	5′-GTCTACACCCCTAACTGCG-3’	60
	Reverse	5′-CTCTGTTGGTCTCTGCGG-3’	
IGFBP7	Forward	5′-GCCCAGAAAAGCATGAAGTAAC-3’	60
	Reverse	5′-TTTATAGCTCGGCACCTTCAC-3’	
ITGA4	Forward	5′-AGAGAGACAATCAGTGGTTGG-3’	55
	Reverse	5′-TCAGTTCTGTTCGTAAATCAGG-3’	
ITGB7	Forward	5′-AGCAGCAACAACTCAACTGG-3’	55
	Reverse	5′-TTACAGACCCACCCTTCCTCT-3’	
FAK	Forward	5′-GAAGCATTGGGTCGGGAACTA-3’	55
	Reverse	5′-CTCAATGCAGTTTGGAGGTGC-3’	
GAPDH	Forward	5′-TCCTTCTGCATCCTGTCAGCA-3’	58
	Reverse	5′-CAGGAGATGGCCACTGCCGCA-3’	

### Flow cytometry analysis

For immunophenotyping of cell surface antigens, third-passage PD-MSCs^PRL-1^ were detached, stained with antibodies conjugated with fluorescein isothiocyanate (FITC) and phycoerythrin (PE) and analyzed with a FACSCalibur flow cytometer (Becton Dickinson, Franklin Lakes, NJ, USA). The following monoclonal antibodies were used: CD34-PE, CD90-PE, HLA-ABC-FITC, HLA-DR-FITC (BD Bioscience, San Jose, CA, USA), CD13-PE (BioLegend, San Diego, CA, USA), CD105-FITC (R&D Systems, Minneapolis, MN, USA), and HLA-G (Abcam, Cambridge, UK). For each sample, at least 10,000 events were acquired.

### Teratoma formation and histological analysis

Nine-week-old male NOD/SCID mice (Laboratory Animal Research Center, Bungdang CHA Medical Center, CHA University, Seongnam, Republic of Korea) were maintained in an air-conditioned animal house under specific pathogen-free conditions. To investigate teratoma formation, PD-MSCs^PRL-1^ (5 × 10^5^ cells) were directly injected into each testis (TP; *n* = 2). Control mice were not injected with cells (Con; *n* = 2). After 14 weeks, the testes were collected, and all mice were sacrificed. The testes were fixed in 10% neutral buffered formalin and embedded in paraffin. Sections were stained with hematoxylin and eosin (H&E). In all animal experimental processes, protocols were approved by the Institutional Animal Care Use Committee (IACUC) of CHA University, Seongnam, Korea (IACUC-180023).

### Western blotting

Total protein was isolated lysis buffer (Sigma-Aldrich). The protein lysates were separated by 8 to 12% sodium dodecyl sulfate polyacrylamide gel electrophoresis (SDS-PAGE) and transferred to polyvinylidene difluoride (PVDF) membranes, which were then blocked in 5% bovine serum albumin and incubated overnight at 4 °C with the following primary antibodies: anti-phospho-PI3K p110α (1:1000, Cell Signaling Technology, Danvers, MA), anti-phospho-AKT (1:1000, Cell Signaling Technology), anti-phospho-mTOR (11,000, Abcam), anti-phospho-FAK (1:1000, Cell Signaling Technology), anti-PPAR-γ (1:500, Santa Cruz Biotechnology, Dallas, TX), anti-leptin (1:500, R&D systems), anti-TNF-α (1:500, Santa Cruz Biotechnology), and anti-GAPDH (1:3000, AbFrontier, Seoul, Republic of Korea). The membranes were then incubated with horseradish peroxidase (HRP)-conjugated secondary antibodies (Bio-Rad, Hercules, CA, USA), and the bands were detected using an enhanced-chemiluminescence reagent (Bio-Rad).

### Karyotyping analysis

Naïve and PD-MSCs^PRL-1^ analyzed karyotypes, respectively using G-banding techiniques. The karyotype analysis was interpreted according to the International System for Human Cytogenomic Nomenclature (ISCN 2016) and visualized under light microscope (Axioskop2 plus, Zeiss, Germany).

### Human cytokine array

Cell culture supernatants in naïve and PD-MSCs^PRL-1^ were collected and analyzed according to the manufacturer’s protocol using Human Proteome Profiler™ Cytokine Array Kit (R&D Systems).

### Statistical analysis

Data analyses were performed using GraphPrism version 5.0 (GraphPad Software, CA, USA) and statistically significant differences were assessed using two-tailed unpaired Student’s t-test or nonparametric statistical test by Mann-Whitney U and Kruskal-Wallis test at a significance level of less than 0.05. All experiments were analyzed in duplicate or triplicate.

## Results

### Characterization of PD-MSCs modified with the PRL-1 gene

**Fig. 1:**
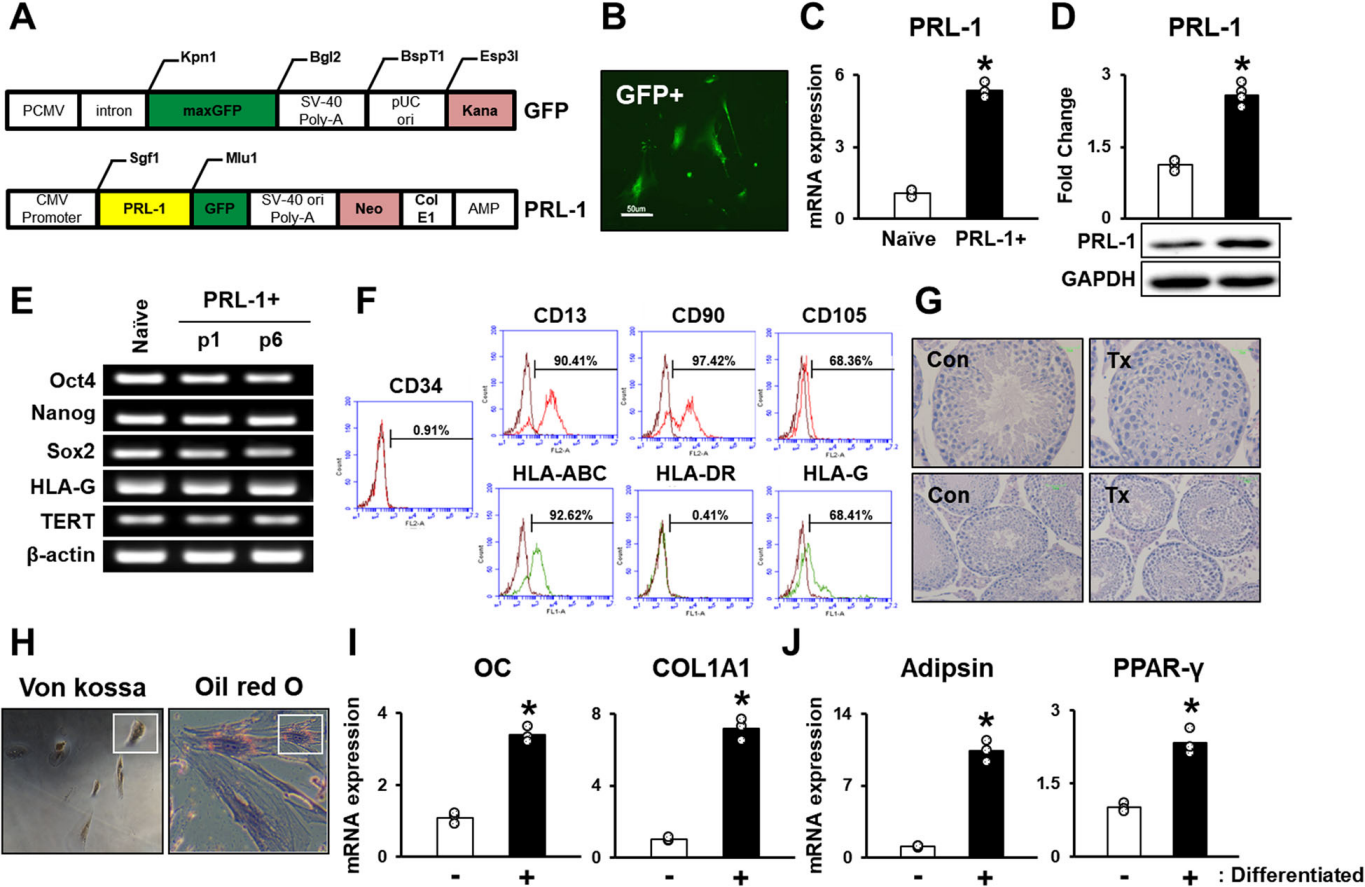
Characterization of PD-MSCs modified with the PRL-1 gene (PD-MSCs^PRL-1^, PRL-1+). **a** GFP and PRL-1 plasmid vector map. **b** Expression of GFP in PD-MSCs^PRL-1^ using a nonviral gene delivery system (GFP+). Scale bars: 50 μm. **c** mRNA and **d** protein expression of PRL-1 in PD-MSCs^PRL-1^ (mean ± SD *p < 0.05 compared with naïve; individual open circle). **e** Stemness markers in naïve PD-MSCs and PD-MSCs^PRL-1^ in cells at different passages were measured by RT-PCR. **f** Hematopoietic, nonhematopoietic, and HLA family surface markers in PD-MSCs^PRL-1^ were measured by FACS analysis. **g** Histopathological analysis of nontransplanted (Con) or PD-MSCs^PRL-1^ transplanted (Tx) into NOD/SCID mouse testes after 14 weeks by H&E staining. **h** Osteogenic and adipogenic differentiations of PD-MSCs^PRL-1^ were assessed using von Kossa and Oil Red O staining. mRNA expression of **i** osteogenic (OC and COL1A1) and **j** adipogenic-specific markers (Adipsin and PPAR-γ) in undifferentiated (−) or differentiated (+) PD-MSCs^PRL-1^ (mean ± SD *p < 0.05 compared with the undifferentiated groups; individual open circle). Statistic significances were determined by nonparametric Mann-Whitney U test

PD-MSCs were transfected with the PRL-1 gene using a nonviral AMAXA system (Fig. 1a). After transfection, PRL-1 expression in PD-MSCs was verified by expression of the GFP reporter gene (Fig. 1b). The mRNA and protein expression levels of PRL-1 in PD-MSCs^PRL-1^ were significantly higher than those in naïve cells (Fig. 1c, d, *p < 0.05). We analyzed the mRNA expression of genes associated with stemness markers (e.g. Oct4, Nanog and Sox2, telomerase reverse transcriptase; TERT, and HLA-G) in PD-MSCs^PRL-1^. As expected, PD-MSCs^PRL-1^ were well maintained at passages 1 and 6 (Fig. 1e). To identify the phenotypes of PD-MSCs, the cell surface markers on PD-MSCs^PRL-1^ were analyzed by flow cytometry. PD-MSCs^PRL-1^ were positive for the expression of the MSC markers CD13, CD90, and CD105 but were negative for the hematopoietic lineage markers CD34 and HLA-DR; however, the HLA class I molecule HLA-ABC was highly expressed (Fig. 1f). Additionally, no teratoma formation was observed after transplantation of PD-MSCs^PRL-1^ (Fig. 1g). Karyotyping of naïve and PD-MSCs^PRL-1^ was diploid and genetically stable (Supplementary Fig. 1). The differentiation into mesodermal lineage was induced in PD-MSCs^PRL-1^. Osteogenic and adipogenic differentiation of PD-MSCs^PRL-1^ were evaluated by positive staining with von Kossa and Oil Red O, respectively (Fig. 1h). We previously confirmed the multidifferentiation potential of naïve MSCs [19]. Osteogenic-specific markers (Osteocalcin; OC and Collagen Type 1 alpha 1; COL1A1) and adipogenic-specific markers (Adipsin and PPAR-γ) were increased in differentiated PD-MSCs^PRL-1^ (Fig. 1i, j, *p < 0.05). These findings suggest that PD-MSCs^PRL-1^ maintain characteristics to those of naïve cells.

### PD-MSCs^PRL-1^ inhibit adipogenesis in OFs from GO patients

**Fig. 2:**
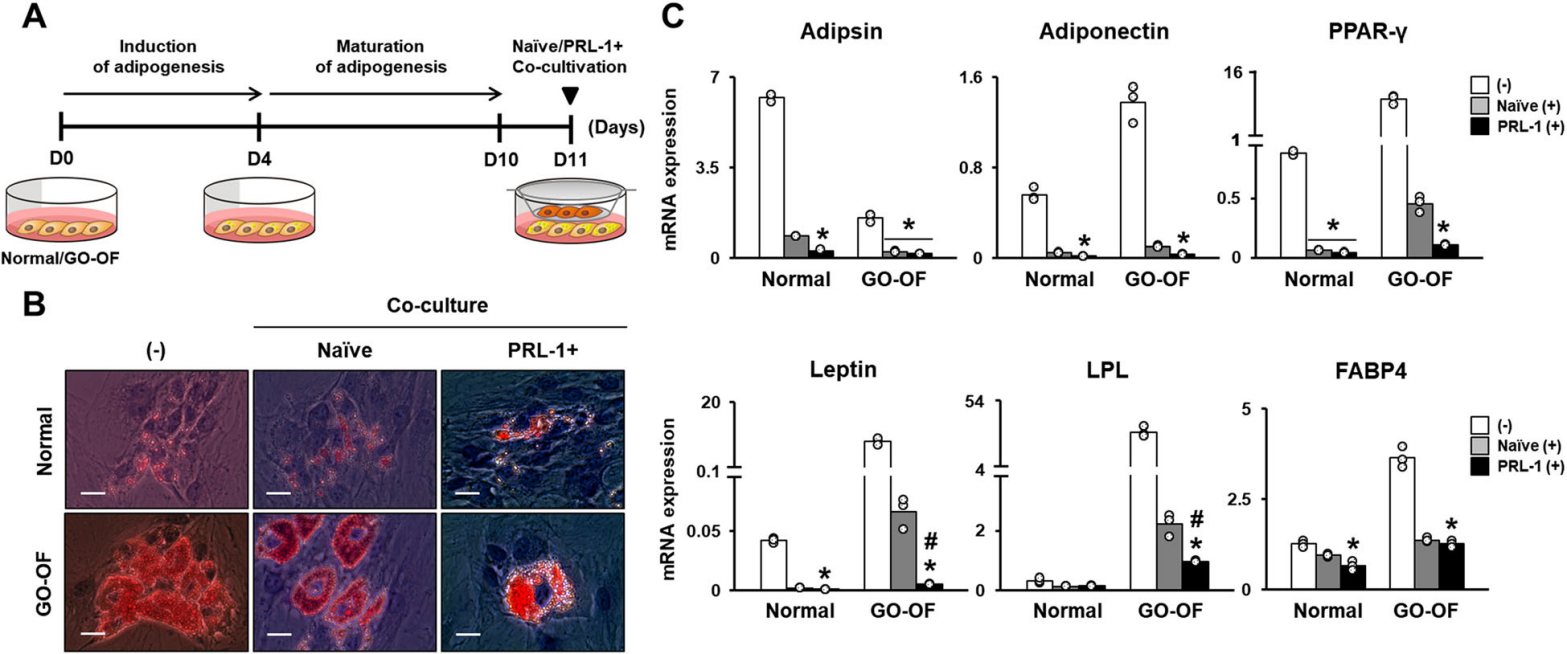
PD-MSCs^PRL-1^ inhibit adipogenesis in OFs from GO patients. **a** Schematic diagram describing naïve PD-MSCs (Naïve (+)) and PD-MSCs^PRL-1^ (PRL-1 (+)) coculture with normal (*n* = 3; individual open circle) and GO-derived OFs (GO-OF; *n* = 3; individual open circle) that underwent adipogenic differentiation. During the first 4 days, adipogenesis was induced in normal and GO-OFs. For 10 days, normal and GO-OFs were maintained and underwent adipogenic maturation. Naïve PD-MSCs and PD-MSCs^PRL-1^ were cocultured in the transwell insert system for 24 h. **b** Representative images of adipogenic differentiation of normal and GO-OFs cocultured with naïve and PD-MSCs^PRL-1^. Scale bars: 100 μm. **c** qRT-PCR analysis of mRNA expression of adipogenic markers in normal and GO-OFs that underwent adipogenic differentiation and were cocultured with naïve PD-MSCs and PD-MSCs^PRL-1^. (mean ± SD *p < 0.05 compared with the noncoculture (−) groups) (mean ± SD #*p* < 0.05 compared with the naïve coculture groups; individual open circle). Statistic significances were determined by nonparametric Kruskal-Wallis test

To evaluate the effects of PD-MSCs^PRL-1^ on adipogenesis in OFs from GO patients, adipogenesis was induced in normal and GO-derived OFs for 4 days, followed by 6 days of maturation. After 10 days of in vitro maturation, differentiated GO-derived OFs were indirectly cocultured with naïve PD-MSCs and PD-MSCs^PRL-1^ (Fig. 2a). Normal and GO-derived OFs were stained using Oil Red O to visualize lipid accumulation (Fig. 2b). The mRNA expression levels of adipogenic-specific markers (e.g. adipsin, adiponectin, PPAR-γ, leptin, lipoprotein lipase; LPL, and fatty acid-binding protein 4; FABP4) in OFs from GO that were cocultured with naïve PD-MSCs and PD-MSCs^PRL-1^ were decreased compared to those of cells that were not cocultured. Interestingly, leptin and LPL expression in cocultured PD-MSCs^PRL-1^ was significantly decreased (Fig. 2c, *p < 0.05, #*p* < 0.05). These findings suggest that PD-MSCs^PRL-1^ downregulate the gene expression of adipogenic markers and inhibit adipogenesis in OFs from GO patients.

### PD-MSCs^PRL-1^ promote the expression of IGFBP genes

**Fig. 3:**
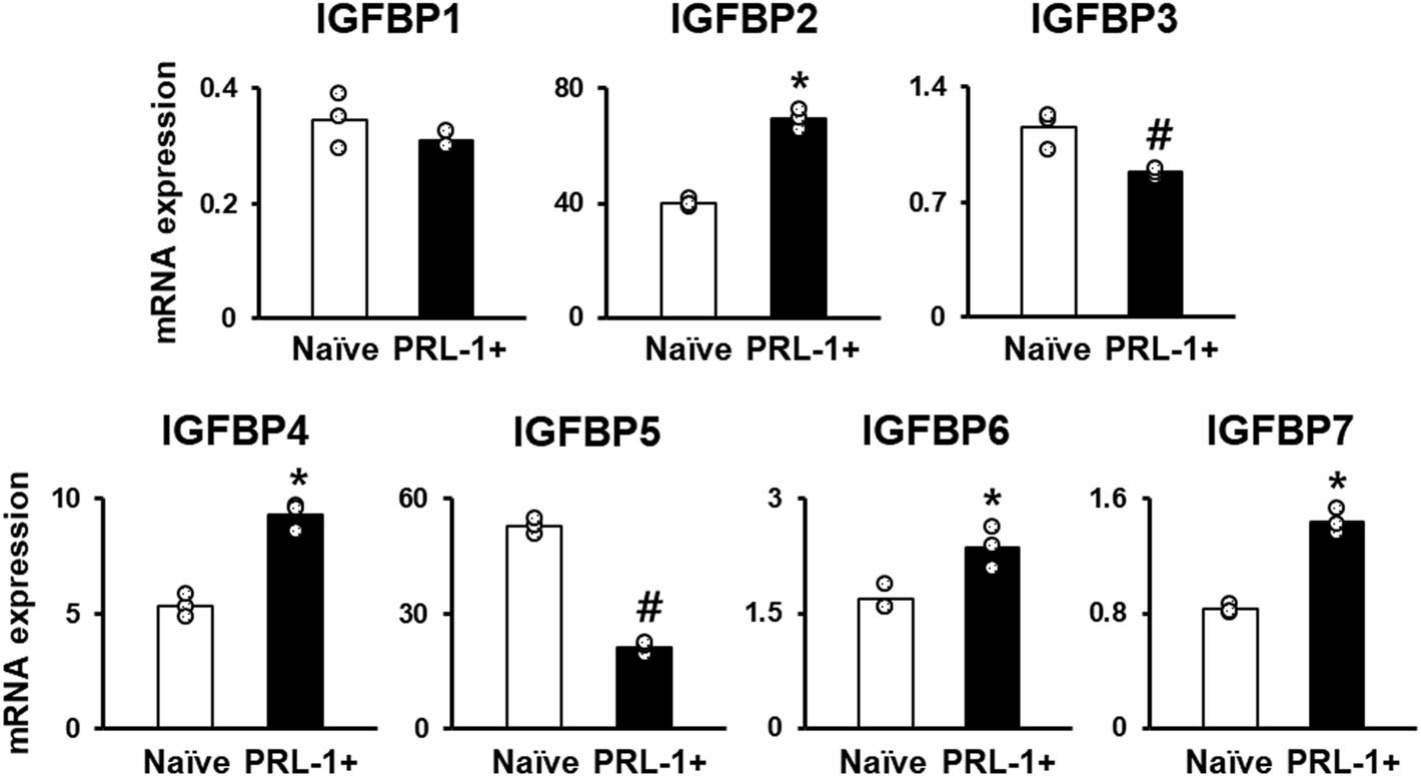
PD-MSCs^PRL-1^ promote the expression of IGFBP genes. mRNA expression of IGFBPs in naïve PD-MSCs and PD-MSCs^PRL-1^ (PRL-1+) were measured using qRT-PCR (mean ± SD *p < 0.05, #*p* < 0.05 compared with naïve; individual open circle). Statistic significances were determined by nonparametric Mann-Whitney U test

IGFBPs control IGF-1R actions by regulating the bioavailability of the ligands IGF-1 and IGF-2 and have been shown to have inhibitory effects on adipogenesis in human visceral adipocytes. We previously analyzed whether naïve PD-MSCs and PD-MSCs^PRL-1^ secreted IGFBP2 and 3 with human cytokine XL profiler arrays (Supplementary Fig. 2). Therefore, we investigated whether PD-MSCs^PRL-1^ promoted the expression of IGFBPs. qRT-PCR analysis revealed that the expression levels of IGFBP-2, − 4, − 6, and − 7 in PD-MSCs^PRL-1^ were significantly elevated, although IGFBP-3 and -5 were reduced compared to those in naïve cells. These data show that PD-MSCs^PRL-1^ upregulate the expression of IGFBP-2, − 4, − 6, and − 7 and may control IGFBP secretion (Fig. 3, *p < 0.05, #*p* < 0.05).

### IGFBPs secreting PD-MSCs^PRL-1^ inhibit adipogenesis via upregulation of FAK and downregulation of the PI3K/AKT/mTOR signaling pathway

**Fig. 4:**
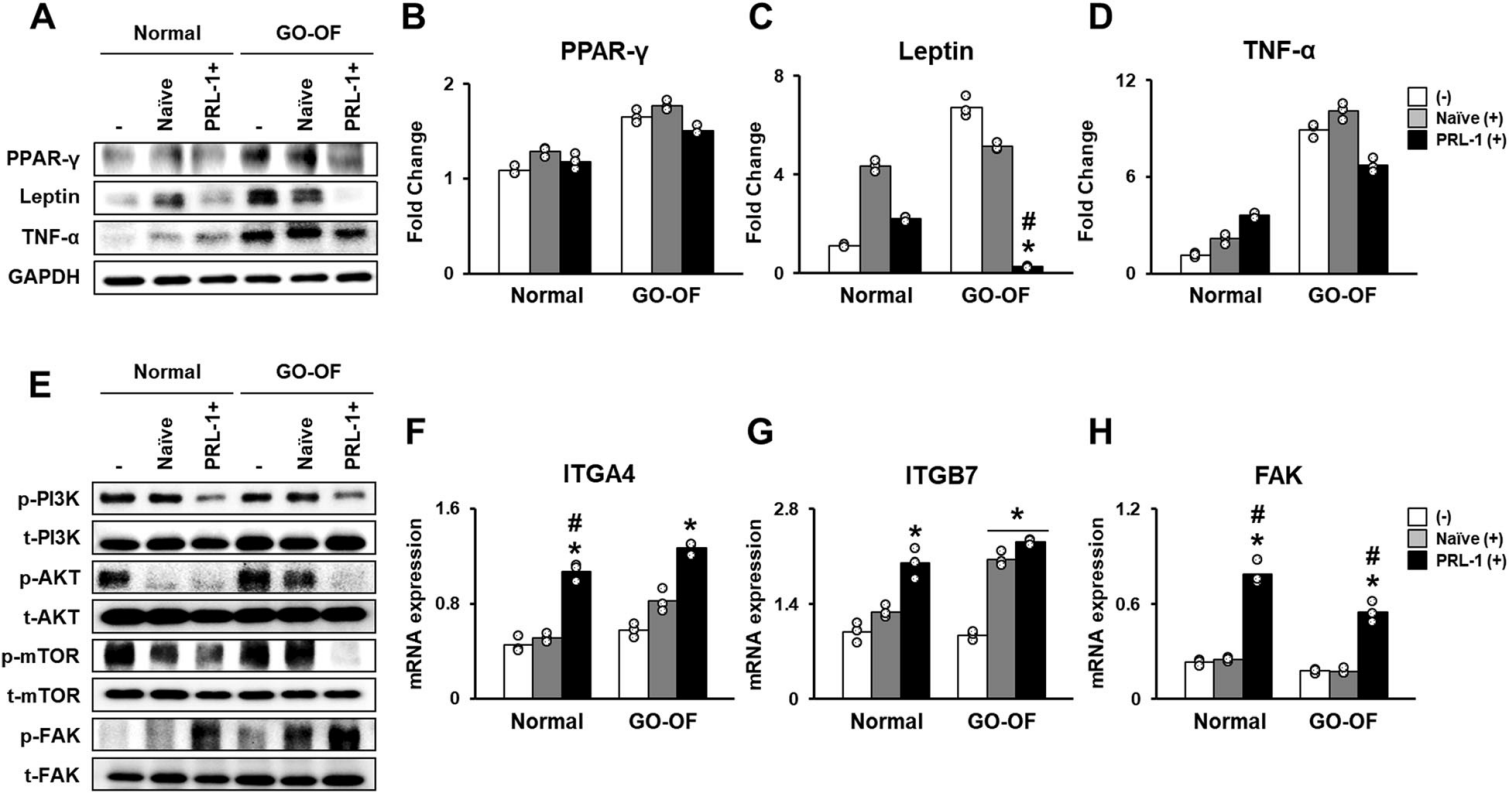
IGFBPs secreting PD-MSCs^PRL-1^ inhibit adipogenesis via upregulation of FAK and downregulation of the PI3K/AKT/mTOR signaling pathway. **a** Protein expression of PPAR-γ, Leptin, and TNF-α in normal and GO-derived OFs (GO-OFs) cocultured with naïve PD-MSCs (Naïve (+)) and PD-MSCs^PRL-1^ (PRL-1 (+)) for 24 h was analyzed using western blotting. Quantitative analysis of **b** PPAR-γ **c** Leptin and **d** TNF-α expression in normal and GO-OFs cocultured with naïve PD-MSCs and PD-MSCs^PRL-1^ for 24 h (mean ± SD *p < 0.05 compared with the noncoculture (−) groups) (mean ± SD #*p* < 0.05 compared with the naïve coculture groups). **e** Protein expression of phosphorylated (p-) and total (t-) PI3K, AKT, mTOR, and FAK in normal and GO-OFs cocultured with naïve PD-MSCs and PD-MSCs^PRL-1^ for 24 h was measured using western blotting. mRNA expression of **f** ITGA4, **g** ITGB7, and **h** FAK in normal and GO-OFs cocultured with naïve PD-MSCs and PD-MSCs^PRL-1^ for 24 h (mean ± SD *p < 0.05 compared with the noncoculture groups) (mean ± SD #p < 0.05 compared with the naïve coculture groups; individual open circle). Statistic significances were determined by nonparametric Kruskal-Wallis test

We confirmed that the increased expression of adipogenic-specific genes in OFs from GO patients was downregulated by PD-MSCs^PRL-1^. Moreover, the protein expressions of PPAR-γ and TNF-α in OFs derived from GO patients cocultured with PD-MSCs^PRL-1^ were decreased compared to that of OFs that were cocultured with naïve PD-MSCs (Fig. 4a, b). Interestingly, leptin expression in the PD-MSCs^PRL-1^ coculture group was markedly decreased compared with that in the naïve coculture group (Fig. 4c, *p < 0.05, #*p* < 0.05). To further investigate the mechanism by which PD-MSCs^PRL-1^ inhibit IGF-1-mediated adipogenesis signaling, we analyzed the expression levels of PI3K/AKT/mTOR pathway members by western blot analysis. The protein expression of phosphorylated PI3K, AKT, and mTOR in OFs from GO patients cocultured with PD-MSCs^PRL-1^ was significantly downregulated compared to that of cells that were not cocultured.

**Fig. 5:**
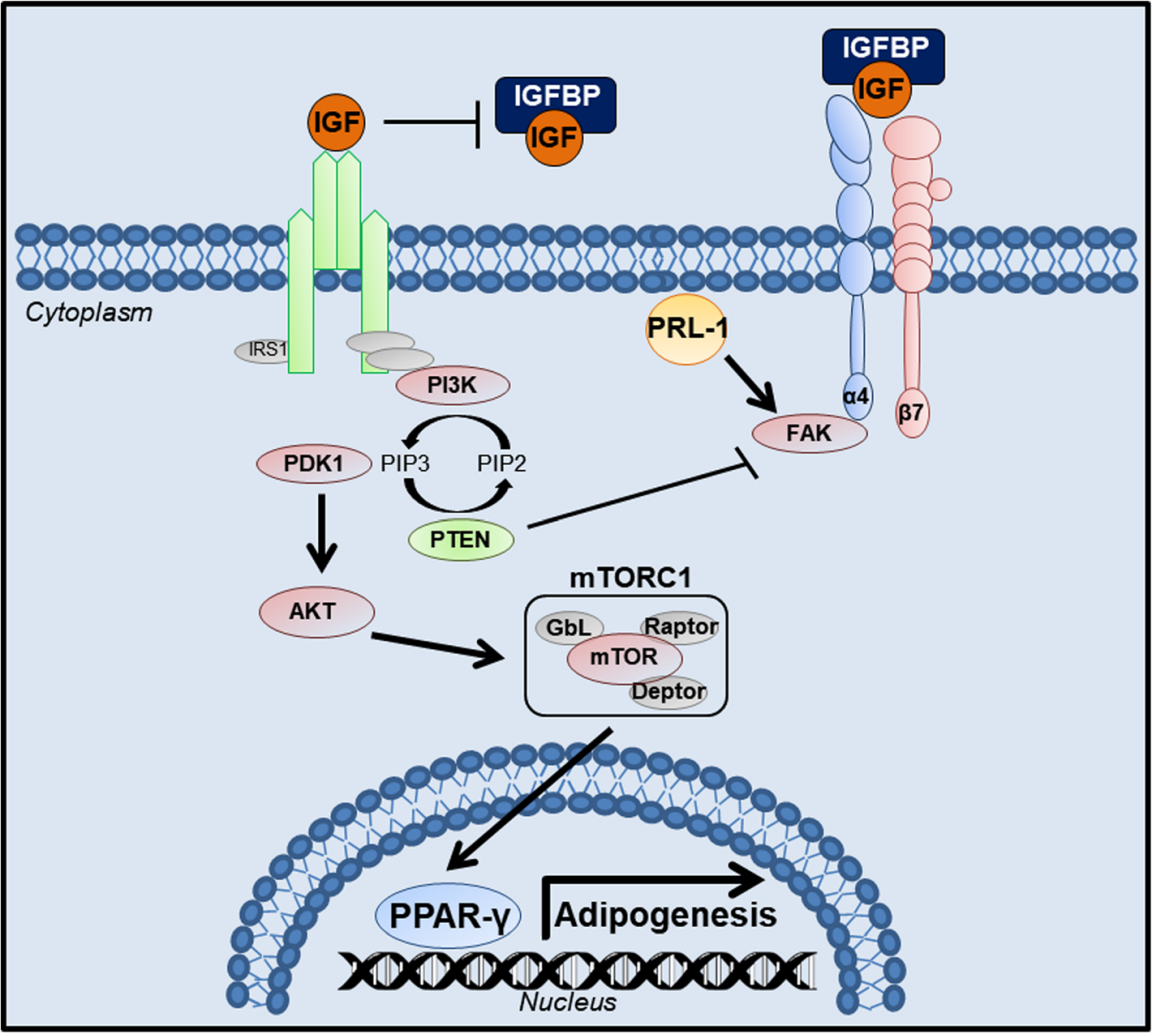
Summarized diagram proposing that IGFBPs secreting PD-MSCs^PRL-1^ are the key antiadipogenic factors inhibiting adipogenesis in OFs from GO patients through upregulation of FAK and downregulation of the PI3K/AKT/mTOR signaling pathway

Interestingly, PD-MSC^PRL-1^ coculture with OFs also decreased the levels of phosphorylated PI3K and the expression of downstream AKT and mTOR compared with those of the naïve coculture group (Fig. 4e). In general, integrins are transmembrane receptors that facilitate cell-extracellular matrix adhesion and can interact with IGFBPs. To confirm that the PD-MSC^PRL-1^-mediated increase in IGFBPs in OFs from GO patients contributes to regulating the adipogenic effect through the integrin signaling pathway, we investigated the expression of ITGA4 and ITGB7 and the integrin downstream signaling factor FAK in normal and GO-derived OFs cocultured with PD-MSCs^PRL-1^. The mRNA expression levels of ITGA4 and ITGB7 in normal and GO-derived OFs cocultured with PD-MSCs^PRL-1^ were higher than those in noncocultured OFs (Fig. 4f, g, *p < 0.05, #*p* < 0.05). Moreover, the mRNA expression of FAK, which is a downstream factor of ITGA4 and ITGB7, in normal and GO-derived OFs cocultured with PD-MSCs^PRL-1^ was significantly higher than that in OFs cocultured with naïve PD-MSCs (Fig. 4h, *p < 0.05, #*p* < 0.05). As shown in Fig. 4e, the mRNA level of FAK was consistent with the protein level. These findings suggest that enhanced IGFBP expression by PD-MSCs^PRL-1^ promotes ITGA4 and ITGB7 signaling, which leads to FAK activation and downregulates the PI3K/AKT/mTOR signaling pathway, resulting in inhibition of OF adipogenesis (Fig. 5).

## Discussion

MSCs have immunomodulatory roles in autoimmune diseases, including GO [20]. Because medical therapies, including corticosteroids and radiotherapy, for patients with GO lead to side effects and the development of malignancies, understanding the molecular mechanisms of de novo adipogenesis and the main symptoms of GO is critical in developing therapeutic applications. In particular, MSCs have anti-inflammatory and immunosuppressive effects on antigen presenting cells and secrete soluble factors, including adipokines, exosomes, and miRNAs [21–24]. Previous reports showed that PD-MSCs have more immunological advantages than other MSCs, as evidenced by the increased expression of HLA-G and the cytokines of IL-2, IL-4, IL-13, and GM-CSF [12]. However, MSC aging results in limited self-renewal abilities and age-associated decreases in cellular numbers and functions [25]. Therefore, gene modification using gene delivery systems overcomes the limited functions of MSCs to provide effective therapeutic results [26]. A recent report revealed that genetically modified MSCs overexpressing IL-35 could be applied in autoimmune diseases to overcome the complications of long-term immunosuppression [27]. Especially, AMAXA technique provides critical opportunities for hard-to-transfect primary cell line including MSCs. In our previous study, we generated TERT-overexpressing PD-MSCs using a nonviral AMAXA system to study the underlying regulatory mechanisms of self-renewal [28]. In addition, PD-MSC^PRL-1^ transplantation in hepatic failure model indicated therapeutic effects including anti-fibrotic and proliferative potentials compared to naïve PD-MSCs [29].

PRL-1 is a member of a subgroup of related protein tyrosine phosphatases contacting a C-terminal prenylation motif [14]. C-terminal residues and cellular redox environments are controlled by the enzymatic activity of PRL-1 [30]. In oxidative-stressed retinas and photoreceptors, modulation of PRL-1 activity regulates redox conditions [16]. We hypothesized that PD-MSCs^PRL-1^ would regulate oxidative conditions and reduce adipogenesis in OFs from GO patients.

Because little is known about the efficacy of PD-MSC^PRL-1^-mediated inhibition of adipogenesis in OFs from GO patients, we further analyzed the functional enhancement of PRL-1 in PD-MSCs generated using a nonviral AMAXA system. OFs isolated from patients with GO are capable of adipocyte differentiation [3]. In orbital adipose tissues and in vitro GO-derived OFs after differentiation, enhanced adiponectin, leptin and PPAR-γ were positively correlated [31]. Previous reports demonstrated that IGF-1 expression was enhanced and PI3K was activated by upregulating PPAR-γ in the orbital fatty connective tissue of patients with GO [32]. In general, IGF binds to IGFBPs. Individual IGFBPs act to increase or attenuate the IGF signaling pathway [33]. IGF-1R/mTOR is associated with differentiation of adipose-derived stem cells (ASCs) [34] and IGFBPs by ASCs regulate the IGF1 effect [35].

Especially, IGFBP2 prevents adipogenesis [36], and IGFBP3 interferes with PPAR-γ-dependent processes to impair adipocyte differentiation [37]. Overexpressed IGFBP2 inhibits both lipogenesis and adipogenesis in visceral adipocytes, and this process involves cell surface association of IGFBP2 and activation of integrin signaling pathway [38]. Similarly, IGFBP4 controls the expression of insulin and IGF1 in mouse adipose tissue expansion [39]. We previously analyzed whether naïve PD-MSCs and PD-MSCs^PRL-1^ significantly secreted IGFBPs using a cytokine array (Supplementary Fig. 2). Based on the results, we found that PD-MSCs^PRL-1^ secreted IGFBP2, − 4, − 6, and − 7 and inhibited adipogenesis. Previously, we confirmed that naïve PD-MSCs have anti-adipogenic effects in the GO animal model [17]. However, our studies are needed to further investigate the molecular mechanism of PD-MSC^PRL-1^ transplantation in animals undergoing experimental GO. In the present study, these findings suggest that IGFBPs secreting PD-MSCs^PRL-1^ through ITGA4- and ITGB7 decreased PPAR-γ-dependent processes via downregulation of PI3K/AKT/mTOR activities and inhibited adipogenesis.

## Conclusions

In this study, we showed that PD-MSCs modified with the PRL-1 gene using nonviral transfection method efficiently overexpressed the PRL-1 protein and maintained the phenotype and multilineage differentiation properties of MSCs. PD-MSCs^PRL-1^ induced IGFBP expression and inhibited adipogenesis via upregulation of FAK and downregulation of the PI3K/AKT/mTOR signaling pathway in OFs from GO patients. In this study, we focused on overcoming the medical problems of GO patients, and functional enhancement of PD-MSCs by nonviral gene modification provides novel insight into next-generation MSC-based cell therapy for future clinical trials in immunological diseases.

## Supplementary Information

**Additional file 1: Supplementary Fig. 1** Karyotyping in naïve PD-MSCs (Naïve) and PD-MSCs^PRL-1^ (PRL-1+).

**Additional file 2: Supplementary Fig. 2** secreted IGFBP2 and 3 in naïve PD-MSCs (Naïve) and PD-MSCs^PRL-1^ (PRL-1+) culture supernatants by cytokine array. Each protein is represented by duplicate spots on the respective membrane (mean ± SD *p < 0.05 compared with naïve).

## Data Availability

All data and materials are available upon request.

## Abbreviations

ASC: adipose-derived stem cells
AMPK: AMP-activated protein kinase
COL1A1: Collagen type 1 alpha 1
FABP4: Fatty acid-binding protein 4
GO: Graves’ ophthalmopathy
HLA: Human leukocyte antigen
HMG-CoA: β-Hydroxy β-methylglutaryl-coenzyme A
IGFBPs: Insulin-like growth factor-binding proteins
IGF-1R: Insulin-like growth factor 1 receptor
ITGA4: Integrin alpha 4
LPL: Lipoprotein lipase
OC: Osteocalcin
OF: Orbital fibroblast
PD-MSCs: Placenta-derived mesenchymal stem cells
PPAR-γ: Peroxisome proliferator-activated receptor-gamma
PRL-1: Phosphatase of regenerating liver-1
TERT: Telomerase reverse transcriptase
TSHR: Thyroid-stimulating hormone receptor
