# Supplementary figures and images for: Functionally enhanced placenta-derived mesenchymal stem cells inhibit adipogenesis in orbital fibroblasts with Graves’ ophthalmopathy
Source: Stem Cell Res Ther. 2020 Nov 5;11:469. doi: 10.1186/s13287-020-01982-3 (PMC7643360; doi:10.1186/s13287-020-01982-3)

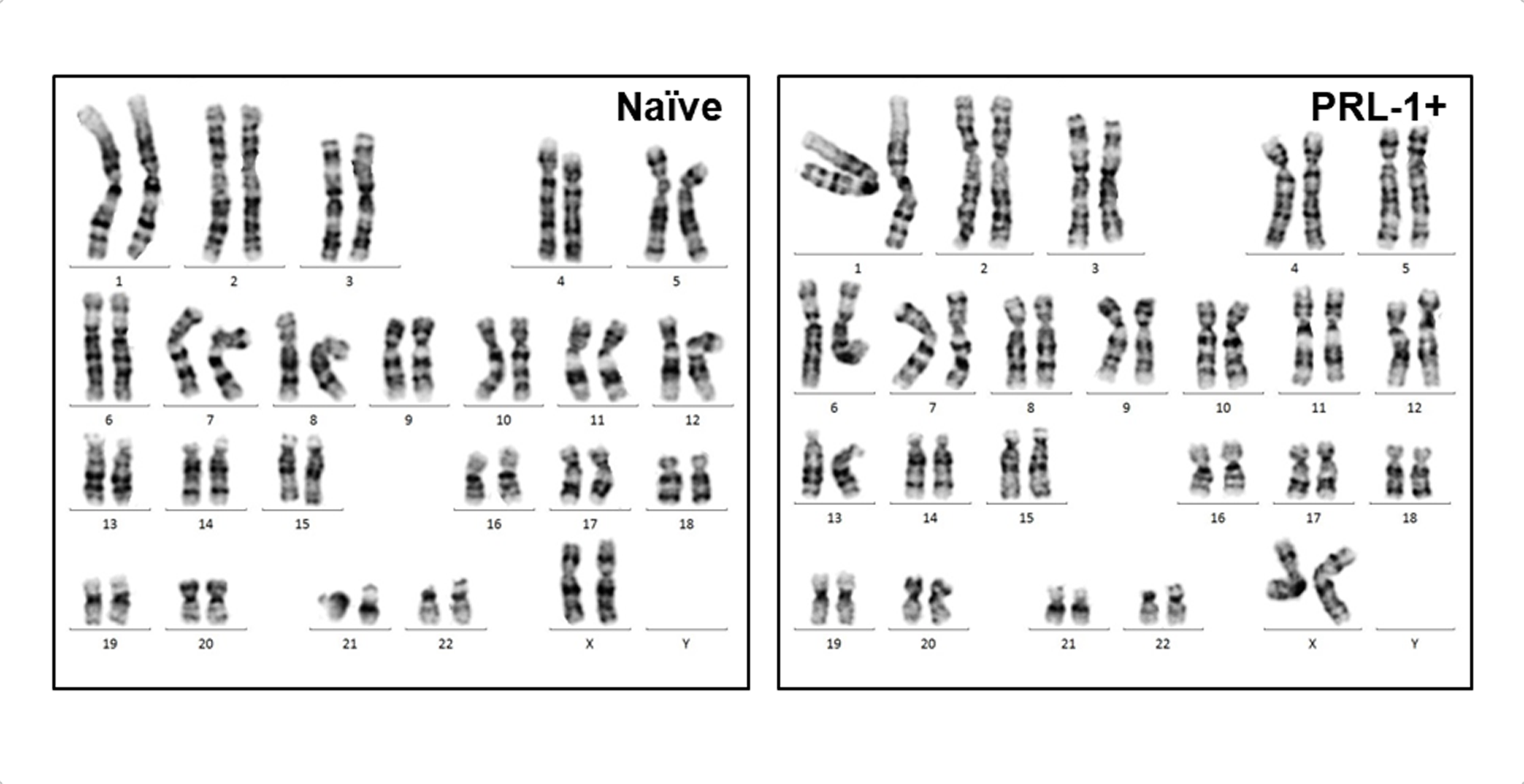

Supplement: Supplementary file 1 — Additional file 1: Supplementary Fig. 1 Karyotyping in naïve PD-MSCs (Naïve) and PD-MSCsPRL-1 (PRL-1+). [file 13287_2020_1982_MOESM1_ESM.tif]

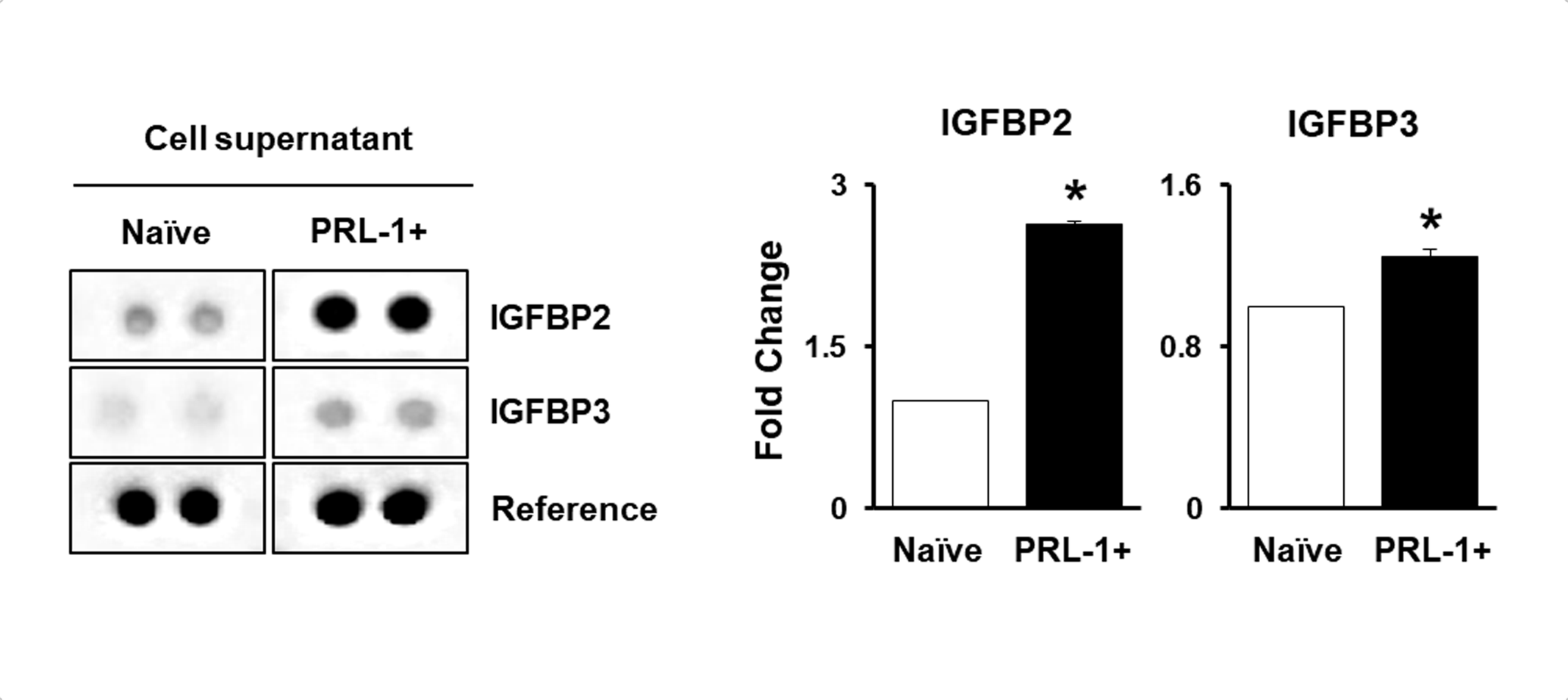

Supplement: Supplementary file 2 — Additional file 2: Supplementary Fig. 2 secreted IGFBP2 and 3 in naïve PD-MSCs (Naïve) and PD-MSCsPRL-1 (PRL-1+) culture supernatants by cytokine array. Each protein is represented by duplicate spots on the respective membrane (mean ± SD *p < 0.05 compared with naïve). [file 13287_2020_1982_MOESM2_ESM.tif]
